# Factors Associated with the HPV Vaccination among Korean Americans and Koreans: A Systematic Review

**DOI:** 10.3390/ijerph19010051

**Published:** 2021-12-21

**Authors:** Soojung Jo, Soo-Yeon Han, Connor A. Walters

**Affiliations:** 1 Edson College of Nursing and Health Innovation, Arizona State University, Phoenix, AZ 85123, USA; soojungj@asu.edu; 2 College of Nursing, The Research Institute of Nursing Science, Seoul National University, Seoul 08826, Korea; 3 School of Social Work, University at Buffalo, Buffalo, NY 14260, USA; cawalter@buffalo.edu

**Keywords:** Korean American, human papillomavirus, vaccination, Asian American

## Abstract

Koreans and Korean Americans (KAs) have limited HPV knowledge and awareness. KAs share a culture with Koreans, and this culture has affected their behavior around HPV. This systematic review aimed to synthesize the factors associated with HPV vaccination among Koreans and KAs. The literature search was done with four databases. The vaccination rate, awareness and knowledge of HPV, and factors associated with vaccination intention were identified. Eighteen articles were selected. Koreans and KAs had low levels of HPV knowledge and awareness. Perceived benefits and seriousness were associated with vaccination intention. Cervical cancer history, beliefs that their daughters need a pap smear test, sexual intercourse experiences, occupation, low education, and income were associated with vaccination intention. This systematic review discovered that HPV vaccination behavior is associated with HPV vaccine awareness, perceived benefits of the vaccine, and the perceived seriousness of HPV infection among Koreans and KAs. Based on the results, we suggest healthcare providers provide a HPV vaccine recommendation by emphasizing the benefits of the vaccination to Koreans and KAs. This study can be the basis for developing interventions to increase HPV vaccination by guiding the target population and variables, as well as the intervention content.

## 1. Introduction

Human papillomavirus (HPV) infection is the most prevalent sexually transmitted infection (STI) in the United States (US) [1]. HPV infection can lead to cervical, vulvar, anal, upper aerodigestive tract, oral, or skin cancer [2]. The cancer most impacted by vaccination, cervical cancer, had an estimated 14,480 new cases in 2021 in the US [3]. Considering the impact of preventing cancer, the vaccination rate is low. Less than half (43%) of females and about a third (31.5%) of males received the HPV vaccine, and this was much lower than other national vaccination programs (more than 88%) [4]. Moreover, research showed the ethnic variations in the vaccination rate: Asian Americans are the lowest HPV-vaccinated population [5]. The percentage of Asian American female young adults who received the vaccine was 22.8%, much lower than the rate for Caucasians (46.3%) [6].

Korean Americans (KAs) are the fifth-largest Asian American subgroup [7], and 1,887,914 were living in the US according to the 2017 US Census Bureau [8]. There is not enough information on the vaccination rate in regard to KAs. However, KAs are the second-most commonly diagnosed with cervical cancer among Asian Americans [9]. The literature has shown KAs had limited HPV awareness compared to Vietnamese and Filipino Americans [10] and limited HPV knowledge [11]. Most KAs do not even know that HPV is an STI [12], and 28% of KA students had never heard of HPV before [13]. Moreover, some KAs do not know HPV can cause cervical cancer [14], while some KAs are more familiar with the term ‘cervical cancer vaccine’ than HPV vaccine [13]. Even if they are aware of HPV, they think the vaccine is only for females [15]. In addition to limited knowledge, other barriers were found, such as healthcare providers not recommending the vaccine to KAs or KAs being worried about side effects, high cost, and not wanting to go to the women’s clinic [14,15,16]. However, little is known about what factors are associated with HPV vaccination behavior in KAs.

In general, immigrant populations are affected by the culture of their original country, especially the culture of their mother [17]. To increase the HPV vaccination rate among KAs, it is essential to investigate HPV knowledge, HPV awareness, and other associated factors, so that healthcare professionals can utilize these factors when they promote HPV vaccinations to these population groups. Foreign-born immigrants are less likely to receive the HPV vaccine than native-born Americans of the same ethnicity (i.e., second or later generations) [18]. Considering KAs inherit their health behavior culture from Korea, their HPV behaviors are similar to Koreans [15]. In Korea, the prevalence of HPV in the general population is estimated at about 20%, but more than 90% of cervical cancer cases are caused by HPV infection [19]. Similar to KAs, the research has reported limited HPV knowledge in Koreans [15,20,21]. More than 90% of Koreans did not know males could be infected by HPV [22]. Even though the HPV national program was started in 2016 for Korean female adolescents, only 67.4% have initiated receiving the first dose of the HPV vaccine [23]. The literature has shown concerns about the cost and adverse effects of the HPV vaccine, which are barriers preventing the receipt of the HPV vaccine among Koreans, as with KAs [21]. As with KAs, no articles at present have systematically reviewed the literature on HPV vaccine behavior in Koreans. Therefore, this systematic review aimed to see what factors are associated with HPV vaccination behavior among KAs and Koreans and synthesize the factors associated with HPV vaccination in this population.

## 2. Methods

### 2.1. Study Selection

A systematic review of the literature was performed, following the Preferred Reporting Items for Systematic reviews and Meta-Analyses (PRISMA) guidelines [24]. We included studies that explore the factors associated with HPV-related knowledge or vaccination among KAs and/or Koreans. We did not limit the basic demographic information (i.e., age or gender) of the population. The inclusion criteria were peer-reviewed journal articles written in English. If we found dissertations, peer-reviewed publications of the dissertations were searched and included. In the intervention studies, baseline data were used for synthesis.

The search databases used were PubMed, CINAHL, PsycInfo, and Web of Science, and we used a combination of the keywords HPV or human papillomavirus AND Korean or Korean American or Asian American from the last five years (from January 2013; the search date was 1 February 2019). An author and a certified librarian searched the literature and screened the initial selection for validation of the inclusion criteria.

### 2.2. Data Collection

From each study, we extracted the theoretical framework, study design, population and basic demographic information, acculturation, the purpose of the study, outcome, implication, and levels of evidence. Two authors reviewed the titles and abstracts for the screening. Full texts were reviewed by the authors independently for study selection.

## 3. Results

**Figure 1:**
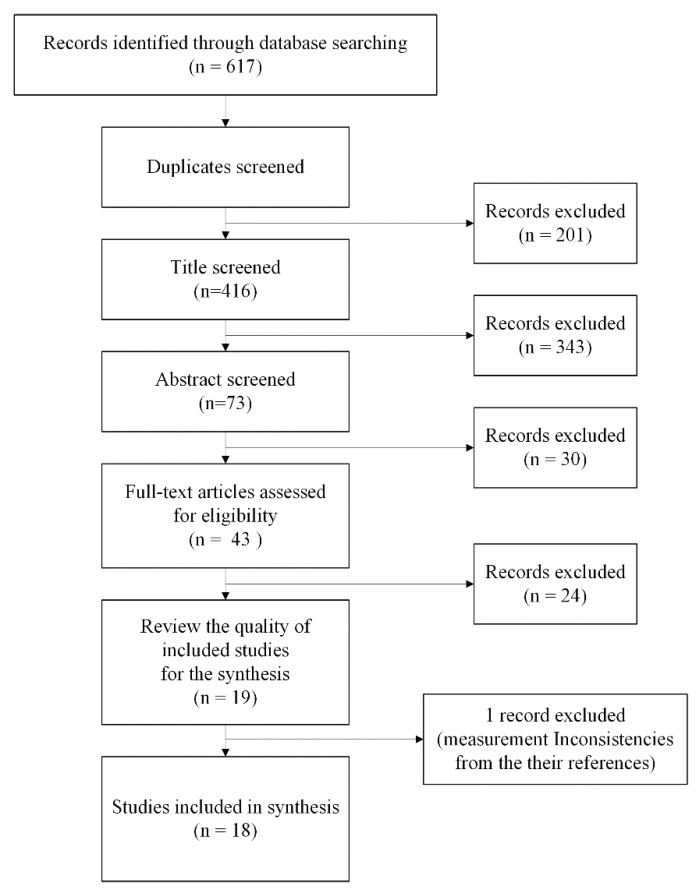
PRISMA diagram of this study (based on Moher et al. 2009).

After the full-text review, 19 studies were found. Figure 1 shows the screening process as a PRISMA diagram [24]. One study was excluded in the synthesis process, because it did not provide enough information on the measurements, and the measurements were not consistent with their original references. Therefore, 18 studies were used for synthesis (Figure 1).

### 3.1. Characteristics of Included Studies

Table 1 shows a summary of the included studies. Most of the studies were quantitative, using a cross-sectional design. Five were qualitative. Two were quasi-experimental. Most of the studies, except four, indicated the data collection year. The range of the data collection was from 2007 to 2016 across 14 studies. Eight studies targeted KAs, and ten studies targeted Koreans. Of the eight about KAs, seven described their inclusion criteria. One study mentioned they recruited first-, 1.5-, and second-generation immigrants [25]. Lee et al. [11] mentioned they recruited the first generation. Lee and Lee [14] described their inclusion criteria as “who was born and grew up in Korea and came to the U.S. as adults, including international college students currently residing in the US”. One study indicated recruiting those who self-identified as Koreans living in the US [12]. Other articles mentioned their inclusion criteria as self-identified KAs. In terms of acculturation questions, the most common questions were years living in the US and English proficiency (preferred language). Questions around cultural identity, associations within the community, or nativity were also asked.

Most studies surveyed only females. Six targeted females and males, and one targeted only males. The targeted age group was mostly adults, accounting for 16 of the studies. Among these 16 articles, the targeted populations included college students, any parents of adolescents, mothers of adolescent daughters, patients who had cervical conization, women who did not take mammograms and/or pap tests in the past two years, women without cervical cancer, school nursing teachers, and general adults. In the studies not targeting adults, one targeted high school and university students aged 15–27, and the other targeted elementary school students aged 11 and 12.

Thirteen studies were based on behavioral theory. The theoretical frameworks used in these were the health belief model (HBM), the information–motivation–behavioral skills model, the behavioral model of health services use, and a self-construal. Three studies combined theories: one used both the HBM and the theory of reasoned action, and two combined the revised network episode model and the theory of planned behavior. Overall, the HBM was the most commonly used; seven studies solely used the HBM, and one used the HBM combined with the theory of reasoned action. The HBM describes health behaviors as being influenced by health belief factors, such as perceived susceptibility, perceived severity, perceived benefits, perceived barriers, cues to action, and self-efficacy [34]. The behavioral construct from the behavior theories will be described in the next sections.

### 3.2. Vaccine Acceptance Behavior

Some studies reported the vaccination rate: roughly 2.4% or 12 out of 495 young men [26], about 6.08% or 73 out of 1200 [32], and around 18.57% or 26 in 140 [31] Koreans and 72.9% of KAs (among 20 people) [16]. The intention of respondents to vaccinate themselves was measured at 25.82% [32] and 58.4% [26] or 3.61 out of five [29]. The intention of respondents to vaccinate their daughters was measured at 70% [31] and 74% [32], which was 64.51 out of 100 [30], and 7.22 out of 10 [33]. The intention to recommend the vaccination for respondents’ students among Korean school health teachers was 5.29 out of 10 [27]. The rate of receiving vaccination recommendations from healthcare providers was 22.1% [13] and 13.13% [31] (Table 2).

### 3.3. Knowledge Level

Most studies measured their knowledge levels. One [11] measured literacy using questionnaires assessing HPV knowledge, so this measure was considered as the knowledge level for the purpose of this review. The measurements of knowledge were varied; each study used different types of measurements. Most studies used yes or no as the answers, but Kim et al. [22] used yes, no, or do not know.

Questions concerning HPV knowledge were about whether HPV infection affects both males and females, the method of transmission of infection, how to prevent HPV, whether HPV can be cured, whether the vaccine is for both males and females, HPV’s relationship with cervical cancer, signs and symptoms of HPV, and ages for the vaccination. The reported knowledge was represented as a percentage of correct answers or the percentage of participants who answered correctly. The range of percentage of correct answers was from 2.2% [22] to 79.8% [13]. The reported correct mean scores were 3.76 [30] and 8.61 [27] out of 13, 1.11 out of 5 [29], 3.14 out of 10 [16], and 4.06 out of 7 [11] (Table 2).

### 3.4. Awareness of HPV

As with the knowledge levels, the awareness of HPV was varied. The percentage of HPV awareness ranged from 1.71% to 86.6%. Awareness of the HPV vaccine was higher than the awareness of HPV itself, ranging from 48% to 92.4%. Two studies asked about awareness of a cervical cancer vaccine rather than an HPV vaccine, and the results ranged from 66.3% [13] to 84.1% [30]. In the studies measuring both the awareness of HPV itself and the HPV and/or cervical cancer vaccine, all the results reported that awareness of the HPV and/or cervical cancer vaccine was higher than awareness of HPV [13,27,30,31]. One study asked three different questions regarding HPV awareness: awareness of HPV, of the HPV vaccine or Gardasil, and of the cervical cancer vaccine. The respondents were highly aware of the cervical cancer vaccine (66.3%), more than of the HPV vaccine or Gardasil (51.9%), less than half of HPV (48.1%; Table 2) [13].

### 3.5. Factors Associated with HPV Vaccination Intention

Six studies analyzed the factors associated with vaccination intention by using multiple regression (Table 3). One study was excluded from the synthesis, because the article did not provide detailed results of the analysis. The population groups from the synthesized results were school teachers, parents of daughters, and the people who were eligible to receive the HPV vaccine. Some studies included variables that were statistically significant in single regression but not significant in multiple regression. While Table 3 includes these variables as a point of interest, our review uses the multiple regression results as a reference point for synthesis. Therefore, the results that were significant in single regression but not in multiple regression were considered insignificant for the purposes of this synthesis.

In terms of the people who were eligible to receive the HPV vaccine, the population was high school students and university students. Age was not a significant factor, but university students had higher vaccination intention than high school students [26]. Sexual intercourse experiences, recommendations by their parents, and perceived benefits were significantly associated with HPV vaccination intentions, but HPV knowledge was inconsistent in two studies: HPV knowledge was significantly associated with the intention to receive the vaccine in KA college women [13], while it was not significantly associated with vaccination intention among Korean high school or university students [26].

Parents, young age, being female, higher educational level, higher income, occupational status, cervical cancer history, belief that their daughter needs a pap smear test, perceived benefits, and perceived seriousness were significantly associated with HPV vaccination intention for their daughters [30,32]. Additionally, young age and higher income, HPV awareness, perceived benefits, and perceived seriousness were associated with HPV vaccination intention for individuals themselves [32]. Regarding school teachers, teachers who were younger and had greater perceived benefits were more willing to recommend the HPV vaccine to their students [27].

## 4. Discussion

This systematic review synthesized the factors associated with HPV vaccination intention in KAs and Koreans. Eighteen articles were utilized for the synthesis, and the findings were as follows.

### 4.1. Low Levels of Knowledge and Awareness

The results showed were variable for HPV awareness. All the studies reported more awareness of the HPV vaccine than awareness of HPV itself. Awareness of the HPV vaccine was especially increased when the researchers asked about awareness of a cervical cancer vaccine rather than HPV or an HPV vaccine. This result is consistent with the results of an earlier study; 96.5% of women know about a cervical cancer vaccine, while only 52% of women had heard about HPV [35]. A possible explanation may be that the HPV vaccine was advertised as a cervical cancer vaccine at the time it was first licensed [36]. There was only one study that surveyed young adolescents who had actually received the vaccine. Only 1.71% of them were aware of HPV, which was remarkably low. This finding aligns with previous study results, though in a different population, that young adolescents have lower levels of awareness than older adolescents [37]. As the HPV vaccination is recommended from age 9, there should be education for adolescents about the vaccine.

In terms of HPV-related knowledge, all the studies reported limited HPV knowledge. The highest correct answer rate was 66.2% from school nursing teachers. It was not possible to compare with other studies that targeted other ethnicities, because each study used different measurements. However, the literature about other Asian Americans has also reported low levels of HPV-related awareness and knowledge in this population [10,38] and that they obtained information about HPV mostly from the media or family members, not from their healthcare providers [10]. This might be characteristic of immigrants as a population generally and applies to Korean Americans as well. The limited knowledge in KAs and Koreans from this study, in combination with the lower awareness shown, suggests the necessity of education from healthcare providers about HPV for KAs and Koreans.

### 4.2. Factors Associated with HPV Vaccination Intention Based on the HBM

The significant factors associated with HPV vaccination intention were young age, being female, higher education, higher income, occupational status, sexual intercourse experience, cervical cancer experiences, beliefs that their daughters need a pap smear, awareness of the HPV vaccine, HPV knowledge, recommendations from parents, perceived benefits, and perceived seriousness. The significance of young age, being female, higher education, higher income, and occupational status were the same as previous studies of Americans and immigrants in America [18]. It is assumed that people with higher education and higher incomes are more likely to have access to HPV information or the healthcare system, so they have a higher intention to vaccinate themselves or their children. One thing to consider is that mothers were more likely to vaccinate their daughters than fathers. Fathers of KAs were not aware of whether or not their children received the HPV vaccine, because mothers have a dominant role in caring for their children [15]. The significant association between HPV vaccination intention and gender might reflect these gender roles. Considering that parental recommendation for the HPV vaccination was an important result in this synthesis, education for fathers is needed as well. As HPV vaccine-related conversations are also linked to sexual topics, KA parents reported finding it challenging to start conversations with their children [15]. HPV infection causes cancers such as anal, upper aerodigestive tract, oral, or skin cancer not only in females but also in males [2]. Hence, sons, as well as daughters, can benefit from the HPV vaccine. A conversation between father and son might be culturally easier than a conversation between mother and son when discussing HPV and the HPV vaccine. Therefore, education that emphasizes HPV vaccination is also proposed to fathers, and as a result, fathers will be able to recommend and persuade their children to get the HPV vaccine.

Among the HPV-related factors, the synthesis of the results in terms of HPV knowledge was inconsistent. Only one study found a significant relationship between HPV knowledge and HPV vaccination behavior, while two studies found a nonsignificant relationship. HPV knowledge was measured using different measurement scales in each study. The inconsistent results might be a result of using different measurement scales. Using different measurement scales could lead studies to be noncomparable. There are some studies that have developed measurement scales of HPV knowledge using a standardized development process [39,40]. Since these predeveloped scales target certain types of age groups, future studies are recommended to use standardized measurement scales for the relevant population to enable comparisons of the results of a specific group with other studies.

HPV vaccine awareness was a significant factor for HPV vaccination intention, while the awareness of HPV itself was not a significant factor for HPV vaccination intention. A possible explanation may be that the HPV vaccine was advertised as a cervical cancer vaccine at the time it was first licensed [36]. Awareness of the HPV vaccine as a cervical cancer vaccine could prevent males from being vaccinated. In addition, even for females, cervical cancer is not the only cancer caused by HPV. Therefore, an educational campaign to promote knowledge of HPV itself, as well as its related cancers, is recommended.

Among the behavior-related factors, perceived benefits and perceived seriousness were significantly associated with HPV vaccination intention. In particular, all the studies that measured perceived benefits reported the significance of this variable’s relationship with HPV vaccine intention. Three studies targeted Koreans, analyzing high school or university students, school teachers, or mothers. This might imply Koreans will receive the HPV vaccine when they receive information about the benefits of the HPV vaccine. Additionally, the HPV vaccine was not yet free at the time the surveys were conducted, so the results of this study could emphasize the importance of providing information on the vaccine’s benefits. This could have an even greater relationship with vaccination now that the HPV vaccine is freely available to adolescent Korean women. One study conducted after the free national HPV vaccination program [32] reported a relationship with perceived benefits, but it did not use a multivariate analysis. Rather, they reported that awareness of the national HPV vaccination program was significantly associated with vaccination intention. Thus, future research to examine the relationship between perceived benefits and awareness of the national HPV vaccine program will allow healthcare providers to adjust the educational content when recommending HPV vaccines.

### 4.3. Quality Issues

While reviewing the results of the included studies, we found that some studies reported a discrepancy in results between the manuscript and tables. Where there was a discrepancy between the article itself and its tables, this study followed the results from the manuscript. Moreover, one article included incorrect information about their measurements. Future studies have to consider the quality of manuscripts by writing and reviewing articles more carefully.

In addition, studies about KAs did not clearly state their definition of KAs. Most studies indicated their inclusion criteria as being a self-identified KA or including both first- and second-generation KAs. There was no limitation for the years of living in the US or their age when they arrived in the US as inclusion criteria. Immigrant generation can be classified in various methodologies such as age at arrival in the US, nativity, or parents’ ethnicity [17]. Moreover, as parental recommendation is more important in the KA population than the recommendation of doctors, the second generation might have different behaviors than the first generation. Thus, future studies for KAs need to apply strict acculturation inclusion criteria and report the results by each generation in order to account for differences among the subgroups of KAs.

### 4.4. Implications

HPV vaccination is the primary preventive measure against HPV infection and its development into cancers. However, racial disparities among HPV vaccination have been reported [6]. While the previous literature has discovered HPV knowledge influences HPV vaccination and HPV infection in American women [41], there is no clear relationship between HPV knowledge and HPV vaccination intention among Koreans and KAs. On the other hand, the perceived benefits of HPV vaccination were a consistently significant factor among Koreans and KAs. Therefore, campaigns on changing the paradigm from discussions of cervical cancer to various HPV-caused cancers, in addition to providing information on the benefits of the vaccine, are suggested at the national level for Koreans. In terms of KAs, the results suggest involving both fathers and mothers in recommending the HPV vaccine to their children, so both parents can persuade sons, as well as daughters, to receive the vaccination against HPV.

### 4.5. Limitations

The major limitation of this study is the analysis of KAs and Koreans as a whole. The reason we did not exclude either group is that it is likely first-generation immigrants are affected by their original culture and have the unique health-related beliefs of that culture [18]. Further, there were not enough quantitative studies about KAs. In addition, this study did not synthesize the results from the qualitative studies. The five qualitative studies included in this study reported results about HPV vaccine acceptance behavior. However, those results were not comparable, since some studies reported neutral information or results from different points of view in a way that prevented direct comparisons. Comparatively, the results from quantitative studies were able to be synthesized, since they provided statistical results. Considering most studies that targeted KAs were using a qualitative design, future studies using a quantitative design are recommended for research about KAs.

Another limitation in the studies about Koreans was that all the studies except one were conducted before the HPV vaccine became free to female adolescents as one of the national vaccination programs in 2016. The vaccine for male adolescents in Korea is not complementary. Therefore, future studies are recommended to examine the attitudes regarding the HPV vaccine and HPV knowledge and see how these have been changed in comparison to the previous studies, as well as how they may differ by sex.

## 5. Conclusions

This systematic review identified the behavioral aspects for HPV vaccination and what factors are associated with HPV vaccination intention in KAs and Koreans. In general, KAs and Koreans have low levels of knowledge and awareness about HPV. While HPV knowledge and awareness had mixed significance across the studies, perceived benefits had consistent results that were significant to HPV vaccination intention. As well as perceived benefits, some demographic factors such as cervical cancer experience or sexual intercourse experience were significantly associated with HPV vaccination intention. The awareness of HPV itself was not a significant factor; on the other hand, awareness of the HPV vaccination was a significant factor.

## Figures and Tables

**Table 1:** Characteristics of the included studies.

Author	Study Design	Koreans or KAs (Definition of KA)	Participants	Year Conducted	*N*	Gender	Age (Mean ± SD)	Theoretical Framework
Choi and Park [26]	A cross-sectional	Koreans	High school and university students	2014	495	Male	15–26 ^a^ (18.4 ± 3.0)	HBM
Choi et al. [27]	A cross-sectional	Koreans	School health teachers (nurses) in elementary, middle, or high school	2011	119	Unknown	24–59 (42.1 ± 9.1)	HBM
Jun et al. [20]	A cross-sectional	Koreans	Cervical conization patients	2014	160	Female	(46.3)	None
Kim [22]	A cross-sectional	Koreans	Adults	2009	1668	Both	19–60 (36.2 ± 8.9, Male) (36.8 ± 9.1, Female)	HBMTRA
Kim [28]	Quasi-experimental	Koreans	Elementary students	2011	117	Both	11–12 (11.9 ± 0.1, Boys) (12.0 ± 0.0, Girls)	HBM
Kim and Kim [21]	Qualitative	Koreans	Mothers of adolescent girls	Unknown	9	Female	Unknown	None
Kim et al. [29]	Quasi-experimental	Koreans	University students	2010	59	Both	(20.5 ± 1.9, Male) (19.7 ± 1.2, Female)	IMB
Kim and Kang [30]	A cross-sectional	Koreans	Mothers of daughters	2011	113	Female	30–67 (44.7 ± 6.2)	HBM
Kim et al. [12]	Qualitative	KAs (Self-identified as Korean female)	Adult women having no mammogram and/or Pap test within the last 2 years	2010–2012	26	Female	36–59 (44.8 ± 6.4)	None
Kim et al. [25]	Qualitative	KAs ((a) first-generation (foreign-born who arrived at the United States when aged 18 years or older), (b) 1.5-generation (foreign-born who arrived at the United States when aged younger than 18 years), and (c) second-generation (born in the United States))	Undergraduate or graduate students	2015	20	Female	18–26 (21.7 ± 2.4)	NEM TPB
Kim et al. [13]	A cross-sectional	KAs (self-identifying as Korean American)	Undergraduate or graduate students	2016	104	Female	18–26 (21.7 ± 2.3)	NEM TPB
Lee and Lee [14]	Qualitative	KAs (who were born and grew up in Korea and came to the U.S. as adults, including international college students currently residing in the U.S.)	Adults	2011	16	Female	21–29 ^b^ (26.0)	NA
Lee et al. [31]	A cross-sectional	Koreans	Mothers of daughters aged 9–14	2015 to 2016	140	Female	Over 30	None
Lee et al. [15]	Qualitative	KAs (self-identified as KA)	Parents of adolescent aged 11–18	Unknown	20	Both	40–53 (46.7 ± 4.1)	HBM
Lee et al. [16]	A cross-sectional	KAs (Not indicated)	Parents of children or adolescents aged 11–18	Unknown	74	Both	(47.2 ± 4.0) ^b^	HBM
Lee et al. [11]	A cross-sectional	KAs (first-generation KA women immigrant)	KA immigrants	2016	235	Female	Over 19	Behavioral Model of Health Services Use
Oh et al. [32]	A cross-sectional	Koreans	Adults	2007 and 2016	1000 in 2007 1200 in 2016	Both	Over 20	HBM
Zhao et al. [33]	A cross-sectional	KAs (Self-identified as Korean American)	Adults without cervical cancer	Unknown	165	Female	(36.7 ± 6.1)	Self-Construal

HBM: The health belief model. TRA: The theory of reasoned action. IBM: The information–motivation–behavioral skill. NEM: The revised Network Episode Model. TPB: The theory of planned behavior. ^a^ There was a difference between the manuscript and abstract. We followed the manuscript. ^b^ There was a difference between the manuscript and table. We followed the manuscript.

**Table 2:** Descriptive characteristics of the HPV-related variables.

	Intention of Vaccination	Vaccination Rate (%)	Awareness (%)	Recommended the Vaccine by Providers (%)	Knowledge	Behavioral Factors
Choi and Park [26]	58.4%	2.4	81.4	-	11.7% (correct answer)	Perceived susceptibility 1.4/4 Perceived severity 1.9/4 Perceived benefits 2.1/4 Perceived barriers 2.7/4
Choi et al. [27]	5.2/10 (to recommend to their students)	-	86.6–92.4	-	8.6/13 (mean score) 66.2% (correct answer)	Perceived susceptibility 3.7/5Perceived severity 3.7/5 Perceived benefits 3.6/5 Perceived barriers 2.8/5 Self-efficacy 4.0/5 Perceived cues to action 3.3/5
Jun et al. [20]	-	-	-	-	13.0–73.0% (of participants)	-
Kim [22]	-	-	-	-	2.2–17.0% (of participants)	Perceived benefits 6.6/10 (Men) 6.8/10 (Women) Perceived barriers 9.3/15 (Men) 8.9/15 (Women)
Kim [28]	-	-	1.7	-	-	-
Kim et al. [29]	3.6/5	-	-	-	1.11/5 (correct answer)	-
Kim and Kang [30]	64.5/100 (to daughters)	-	29.2–84.1	-	3.7/13 (mean score) 28.9% (correct answer)	Perceived susceptibility 2.2/5 Perceived benefits 3.4/5 Perceived barriers 2.6/5 Perceived cues to action 3.1/5
Kim et al. [13]	-	-	48.1–66.3	22.1	8.7–79.8	-
Lee and Lee [14]	-	-	-	-	12.5% (one item)	-
Lee et al. [31]	70.7% (to daughters)	18.5	47.0–48.0	13.1	51.0%	-
Lee et al. [16]		72.9 (of their children)	-	-	3.14/10 (mean score)	Perceived susceptibility 11/20 Perceived benefits 5.8/6 Perceived barriers 2.5/4
Lee et al. [11]	-	-	-	-	4.06/7 (correct answer)	
Oh et al. [32] 2007 data	-	-	-	-	8.6–13.3%	Perceived susceptibility 19.5% Perceived benefits 68.4% Perceived seriousness 32.5%
Oh et al. [32] 2016 data	25.8% (respondents) 74.0% (to daughters)	6.0	-	-	35.8–36.9%	Perceived susceptibility 25.7% Perceived benefits 51.2% Perceived seriousness 39.1%
Zhao et al. [33]	7.2/10 (to daughters)	-	-	-	-	-

**Table 3:** The factors associated with HPV vaccination intention.

	Respondents	Age	Gender	Education level	Higher Income	Occupational Status	Awareness of HPV	Awareness of HPV vaccine	Sexual Intercourse Experiences	HPV Knowledge	Cervical Cancer History	Beliefs Daughters Need Pap Smear	Recommended by Doctors	Recommended by Parents	Perceived Susceptibility	Perceived Severity	Perceived Benefits	Perceived Barriers	Perceived Seriousness
Choi and Park [26]	High school, University Students (Korean)	•						•	√	•					•	•	√	x	
Choi et al. [27]	Teachers (Korean)	√		x			•	x									√		
Kim and Kang [30]	Mothers (Korean)					√		√		x	√	√			x	•	√	x	
Kim et al. [13]	College women (KA)	x					x	•		√			•	√					
Oh et al. [32] ^a^	Adults (Korean)	√ (you-ng)	√ *	√ *	√			√											√

Only the variables that were significant in multiple regression were used for our synthesis ^a^ Article includes multiple sets of data; data in the table is from the 2016 survey. √ Indicates a significant association detected in multiple regression. x Indicates no significant association detected. • Indicates a significant association detected in a single linear regression analysis but no significant association detected in a multivariate regression analysis. * Indicates the factor was significant only for the intention to vaccinate their daughter, not themselves.

## Data Availability

Not applicable.
